# Rethinking Solid-Phase Microextraction (SPME) for Bioanalysis: Workflow Optimisation in Automated Vacuum-Assisted Cooling SPME for Fatty Acid Monitoring in Faecal Waters

**DOI:** 10.3390/molecules31203716

**Published:** 2026-10-16

**Authors:** Lorenza Putignani, Stefano Dugheri, Niccolò Fanfani, Giovanni Cappelli, Ilaria Rapi, Michele Santillo, Giorgio Marrubini, Chiara Marangelo, Pamela Vernocchi, Federica Del Chierico, Maria Rita Braghini, Giulia Andolina, Anna Alisi, Antonio Baldassarre, Nicola Mucci

**Affiliations:** 1 Unit of Microbiomics and Research Unit of Microbiome, Bambino Gesù Children’s Hospital, IRCCS, 00146 Rome, Italy; lorenza.putignani@opbg.net or l.putignani@unilink.it; 2 Department of Life Sciences, Health and Health Professions, Link Campus University, 00165 Rome, Italy; 3 Department of Experimental and Clinical Medicine, University of Florence, 50121 Florence, Italy; 4 Division of Occupational Medicine, Careggi University Hospital, 50134 Florence, Italy; 5 Department of Drug Sciences, University of Pavia, 27100 Pavia, Italy; 6 Research Unit of Microbiome, Bambino Gesù Children’s Hospital, IRCCS, 00146 Rome, Italy; 7 Research Unit of Genetics of Complex Phenotypes, Bambino Gesù Children’s Hospital, IRCCS, 00146 Rome, Italy

**Keywords:** short-chain fatty acids, solid-phase microextraction, gas chromatography–mass spectrometry, microwave-assisted derivatisation, green analytical chemistry, faecal waters, gut microbiota biomarkers

## Abstract

Short-chain fatty acids (SCFAs) are key biomarkers of gut microbiota and intestinal health, yet their analysis is challenging due to high volatility, hydrophilicity, and the complexity of the faecal matrix. This study introduces a novel, green, and fully automated workflow for monitoring SCFAs in faecal water samples. It combines microwave-assisted derivatisation, vacuum-assisted cooling multiple solid-phase microextraction (Vac-HS-cooling-MSPME), and fast gas chromatography–mass spectrometry (GC-MS). On-sample derivatisation with 2,3,4,5,6-pentafluorobenzyl bromide was optimised via design of experiments, reducing reaction time from 3 h to just 2 min at 100 °C while preserving high yields and selectivity. Integration of Vac-HS-cooling-MSPME with a fast GC column (5 m × 0.1 mm ID × 0.4 µm) increased sample throughput from two to approximately six samples per hour, with total chromatographic times of 5.24 min. MSPME specifically improved sensitivity for low-response analytes such as pentafluorobenzyl formate. The method demonstrated excellent linearity (R^2^ > 0.998), low limits of detection (0.002–0.09 mg/L), good intra- and inter-day precision, accuracies exceeding 85%, and consistent recovery for each analyte (86–90%). The AGREE greenness score improved from 0.65 to 0.68, confirming alignment with green analytical chemistry principles. This system offers a high-throughput, sensitive, and eco-friendly tool for SCFA quantification, confirmed by the application on real paediatric faecal water samples.

## 1. Introduction

Short-chain fatty acids (SCFAs) are saturated aliphatic monocarboxylic acids containing fewer than six carbon atoms that are predominantly produced by microbial fermentation of dietary carbohydrates in the gut [1]. Acetate, propionate, and butyrate are generally considered the major SCFAs, while other short-chains fatty acids, including valerate and formate, can also be produced by microbial fermentation products [1,2]. SCFAs contribute to host homeostasis by modulating energy metabolism, immune responses and cellular signalling, and they also influence the growth and composition of the gut microbiota. In the colon, SCFAs, and particularly butyrate, serve as the primary energy source for colonocytes and contribute to overall energy balance and nutrient metabolism.

The type and quantity of SCFAs produced depend on the amount of material available to the microbiota for degradation, as well as on the composition of the microbiota itself. Acetate, propionate and butyrate present an approximate ratio often reported around 60:20:20 (acetate:propionate:butyrate). However, the relative abundance of individual SCFAs can vary substantially depending on dietary substrate availability, gut microbiota composition, intestinal location, host factors and disease status [3]. A clear correlation has been suggested between SCFAs (in terms of both type and quantity) and certain human conditions such as irritable bowel syndrome, diarrhoea or other inflammatory bowel diseases, cancer and neurological disorders [4]. This highlights the need to precisely identify the SCFAs that may be responsible and to analyse them on a daily basis in laboratories. One challenge relates specifically to the anatomical location, as the human proximal colon, where SCFA biosynthesis takes place, is difficult to access; furthermore, as SCFAs are rapidly absorbed by the colonic mucosa, it is complicated to quantify them. Fortunately, measuring short-chain fatty acids following colonic transit may offer a non-invasive method for exploring the relationships between these acids and the gut microbiota or gut-related diseases. This approach would help to evaluate fatty acids as indicators of health.

Several analytical approaches have been developed for the determination of SCFAs in faecal samples, including gas chromatography coupled with flame ionisation detection (GC-FID) or mass spectrometry (GC-MS) [5,6,7], high-performance liquid chromatography (HPLC) [8], ultra-high-performance liquid chromatography coupled with tandem mass spectrometry (UHPLC-MS/MS) [9], capillary electrophoresis [10], and headspace solid-phase microextraction (HS-SPME) coupled with GC-MS. GC-based methods are widely used for SCFA analysis because of their sensitivity. Zhao et al. described a rapid procedure based on acidified aqueous extraction and direct-injection GC for the determination of SCFAs in human and rat faeces [11], while Zhang et al. developed a headspace-GC method for the analysis of SCFAs in faecal samples [12]. More recently, solid-phase extraction and simplified extraction procedures have also been proposed for the quantification of faecal SCFAs [13,14]. HS-SPME coupled with GC-MS or GC-MS/MS has further been investigated as a miniaturised approach, although analytical performance can be influenced by factors such as pH, salting-out conditions and fibre characteristics [15]. Despite the availability of several analytical procedures, the determination of SCFAs in faecal matrices remains challenging because of their volatility, polarity and the complexity and heterogeneity of the biological matrix. Derivatisation strategies can improve chromatographic behaviour and detection sensitivity [5,9], whereas miniaturised extraction techniques such as HS-SPME may reduce sample handling and solvent consumption [15]. GC-MS methods commonly employ 2,3,4,5,6-pentafluorobenzyl bromide (PFBBr) derivatisation to improve SCFA detectability [16]; however, said procedure is often time-consuming and presents drawbacks related to reagent corrosiveness, background noise, and controlled heating conditions [17]. Microwave-assisted derivatisation (MAD) has been proposed as an alternative to conventional heating, exploiting the interaction of microwave radiation with polar or ionic species to accelerate and homogenise the derivatisation reaction [18,19,20], being applied to several analytical and derivatisation procedures, offering potential advantages in terms of reaction time, efficiency, and throughput [21,22]. Vacuum-assisted HS-SPME has been investigated to enhance analyte transfer [23], while cooled SPME, based on simultaneous sample heating and headspace or fibre cooling, can further improve extraction efficiency and sensitivity [24,25,26], since conventional SPME procedures may still require relatively long equilibration and extraction times.

Faecal water, the aqueous or soluble fraction of faecal material that contains a complex mixture of metabolites and other soluble compounds, is also used as an experimental matrix to investigate the biological effects of soluble faecal components on intestinal epithelial cells [27]. Previous studies have exposed Caco-2 cells to faecal water to investigate cellular toxicity and the effects of dietary components, with SCFAs considered among the faecal metabolites potentially contributing to these effects [28]. However, based on the literature identified in the present search, direct studies specifically quantifying SCFAs in faecal water before and after exposure to Caco-2 cells appear to be limited.

We propose a novel fully automated vacuum-assisted headspace cooling multiple solid-phase microextraction (Vac-HS-cooling-multiple-SPME) method for SCFA monitoring of faecal waters, after 2,3,4,5,6-pentafluorobenzyl bromide (PFBBr) on-sample derivatisation by microwave-assisted derivatisation (MAD), analysed by GC-MS. A rapid multiple-SPME configuration using the same fibre for parallel extraction from two aliquots of the same sample (“single-fibre MSPME”) has been developed, reducing extraction times to less than 6 min and strongly enhancing the throughput. To our knowledge, this is the first application combining Vac-HS-cooling-multiple-SPME and MAD for SCFA analysis in faecal waters. Thanks to design of experiments (DoE), the number of experiments required during the experimental development phase is reduced, thereby helping to ensure compliance with green analytical chemistry (GAC) principles. The environmental sustainability of the proposed method for quantifying SCFAs is assessed using the Analytical GREEnness tool [29], where the method’s performance is compared with that of existing procedures to offer a more effective alternative for the biological monitoring of faecal water. Faecal water is generally obtained by separating the aqueous phase from the solid faecal matrix, either directly or following extraction with an aqueous buffer such as phosphate-buffered saline (PBS) [30,31]. Therefore, as a preliminary biological application, the analytical method was applied to faecal water obtained from paediatric subjects with metabolic dysfunction-associated steatotic liver disease (MASLD) and controls, before and after exposure to Caco-2 cells. This experimental setting was intended as a preliminary assessment of changes in SCFA concentrations following contact of the faecal-water-containing medium with intestinal epithelial cells, in order to characterise SCFA stability during the incubation and changes associated with potential cellular uptake and handling, rather than as a model of microbial SCFA production.

## 2. Results and Discussion

In the sample-preparation procedure, 1 g of faeces was initially diluted with 10 mL PBS to obtain faecal water; subsequently, 250 µL of faecal water was diluted to 1 mL before derivatisation and analysis. Therefore, assuming quantitative transfer and neglecting extraction losses, the investigated calibration interval corresponds approximately to 40–5000 mg/kg wet faeces. This conversion provides a more appropriate basis for comparison with clinical studies reporting SCFA concentrations per unit mass of faeces. This correspondence is particularly relevant for the paediatric population investigated in the present study. For example, published data in children with normal body weight and obesity reported faecal concentrations with ranges of 0.026–3.269 mg/g for acetate, 0.050–2.128 mg/g for propionate, 0.030–0.948 mg/g for butyrate, 0.010–0.484 mg/g for formate and 0.063–0.472 mg/g for valerate [32]. The corresponding concentrations in the final analytical solution, considering the 1:40 dilution factor described above, are approximately 1.04–130.8 mg/L for acetate, 2.0–85.1 mg/L for propionate, 1.2–37.9 mg/L for butyrate, 0.4–19.4 mg/L for formate and 2.52–18.9 mg/L for valerate. Thus, these published paediatric faecal concentrations are encompassed by the 1.0–125.0 mg/L calibration interval for the investigated analytes, with the exception of concentrations below 1 mg/L, which remain covered by the reported LOQs of the method (see Section 2.6). However, for the faecal water samples obtained in our experiment there are no reference values, even though there is little evidence that SCFA levels in faecal water are significantly lower [28,33,34].

### 2.1. Overview of Analytical and Microextraction Techniques

For SCFA analysis, GC-MS offers high sensitivity and separation power but generally requires derivatisation, while LC-MS is a versatile alternative but relies on expensive equipment and, in most methods, also requires time-consuming derivatisation with significant variability [35]. Method selection depends on the target SCFAs, detection limits, and available resources. GC-MS with prior derivatisation is widely used [16], particularly with PFBBr. Although derivatisation can be replaced by sialylation, PFBBr improves the detection of compounds reacting with halogenated agents. Its main drawbacks are reagent corrosiveness, background noise under extreme conditions, and long reaction times, especially for large biomonitoring samples [17]. MAD can accelerate the reaction through efficient internal heating and non-thermal effects, improving yields, product purity, and reducing interference [18,19,20]: it has therefore become increasingly used in organic synthesis and quantitative derivatisation [21,22], with commercial systems improving reproducibility, safety, and throughput.

Sample pretreatment remains a bottleneck in GC and LC, driving the development of resource-saving microextraction techniques (METs), defined by IUPAC as techniques using an extractant volume substantially smaller than the sample volume [36]. SPME avoids polluting organic solvents and enables automated, stable, and reproducible sampling, including FFA SPME fibres [32]. These approaches align with GAC principles through miniaturisation, greener solvents, and more efficient preparation, supported by tools for sustainability assessment [37,38], chromatographic modelling [39], and robotic autosamplers [40]. In SPME, analytes partition among the sample, headspace, and fibre. Vacuum-assisted Vac-HS sampling can increase VOC transfer to the HS [23], although few studies have investigated this approach for liquid samples [24]. Heating the sample while cooling the support (SPME cooling) improves extraction and sensitivity, particularly in complex matrices [25]. Cooling can be internal or external; this study uses external cooling with circulating liquids [26]. Equilibration typically requires 30–60 min, while shorter extraction times reduce recovery and increase detection limits. To overcome this limitation, “single-fibre MSPME” uses the same fibre for two aliquots, reducing extraction time to under 5 min. The authors therefore investigated fully automated Vac-HS-cooling-multiple-SPME sampling of SCFAs after on-sample PFBBr derivatisation by MAD and GC-MS analysis as an alternative to conventional methods requiring long derivatisation times [41]. Each step was optimised and compared with a conventional GC-MS method before application to biological samples.

### 2.2. PFBBr Derivatisation

Although SCFA profiling is widely used to study gut bacteria [42], their high volatility and hydrophilicity, together with the heterogeneity of biological matrices such as faeces, make chromatographic analysis challenging. PFBBr derivatisation of carboxyl groups (RCOO^−^) is effective [43], but is limited by reagent corrosiveness, high background noise, high reaction temperatures, and the need for a well-ventilated facility because PFBBr is a tear gas. The main challenge is therefore optimising derivatisation time and temperature to balance volatility, thermal stability and sensitivity against prolonged reactions and by-product formation. Few standard protocols define optimal conditions for individual or comprehensive analytes, although catalysts and alternative heating mechanisms have been investigated to improve GC derivatisation [44]. MAD provides comparable or better results in significantly shorter times and is particularly suitable for PFBBr derivatisation, which can require up to 3 h [41].

**Figure 1:**
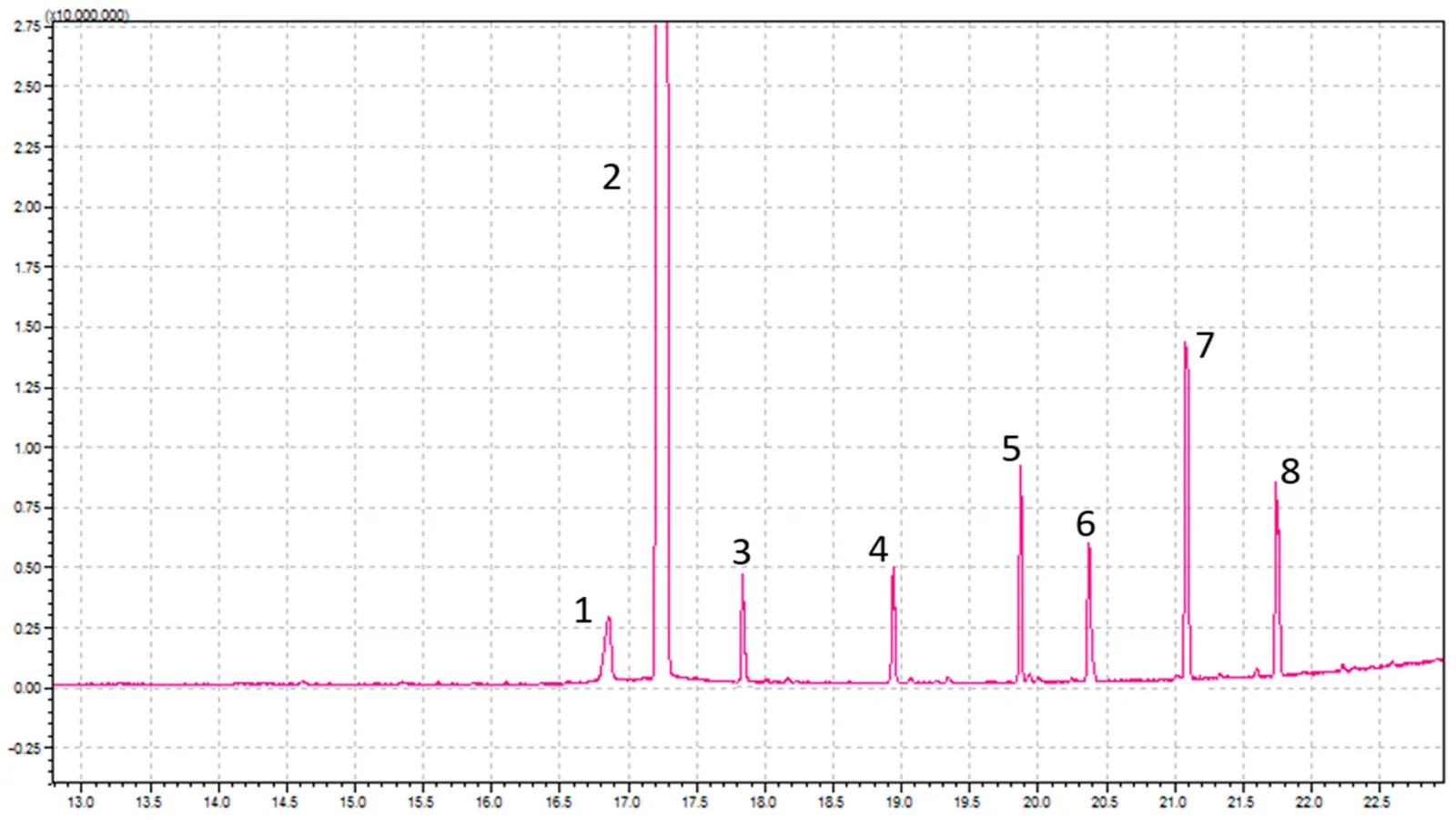
Chromatogram in TIC mode obtained using VF-5 60m column for SCFAs determined (100 mg/L standard). 1. Formic derivative; 2. residual PFBBr; 3. acetic derivative; 4. propionic derivative; 5. butyric derivative; 6. isovaleric derivative; 7. IS derivative; 8. hexanoic derivative.

SPME applied to PFBBr-derivatised solutions can reduce PFBBr-related noise and corrosive effects while improving detection of derivatives. Because excess PFBBr interfered with chromatographic separation, on-sample derivatisation was selected, despite the strong influence of pH on SCFA derivatisation in aqueous solution. Preliminary tests at pH 4, 5, 6, 7, 8 and 10 showed maximum total acid extraction at pH 6; however, while formic acid-PFBBr reached maximum intensity at pH 6, other SCFAs showed low intensities. Since the intensity difference between neutral and alkaline conditions was <10% for all derivatives, pH 7 was selected as optimal. Derivatisation proceeds through nucleophilic displacement of Br^−^ from PFBBr by RCOO^−^. At pH 8–10, excess OH^−^ promotes PFBBr hydrolysis rather than fatty-acid esterification. PFBBr-SCFA mass spectra showed an intense m/z 181 fragment corresponding to [C6F5-CH2•]+ and m/z [M-181], plus m/z 161 from HF loss. The most abundant ions were m/z [M-197] and [M-225], corresponding to loss of C6F5-CH2-O and C6F5-CH2-O-CO, respectively. All SCFA-PFB derivatives showed the molecular ion (M^+^) at 1–20% abundance. Figure 1 reports the chromatogram obtained with the conventional GC derivatisation method. As can be seen, no unreacted SCFAs were detected in their elution region (13–17 min, previously determined by the authors in survey tests employing shorter derivatisation times). The peak of residual PFBBr is present at 17.25 min, while formic, acetic, propionic, butyric, isovaleric and hexanoic derivatives can be observed at 16.85, 17.85, 18.92, 19.85, 20.35 and 21.75 min, respectively. The IS derivative peak can be observed at 21.10 min.

### 2.3. Chemometric Optimisation

**Figure 2:**
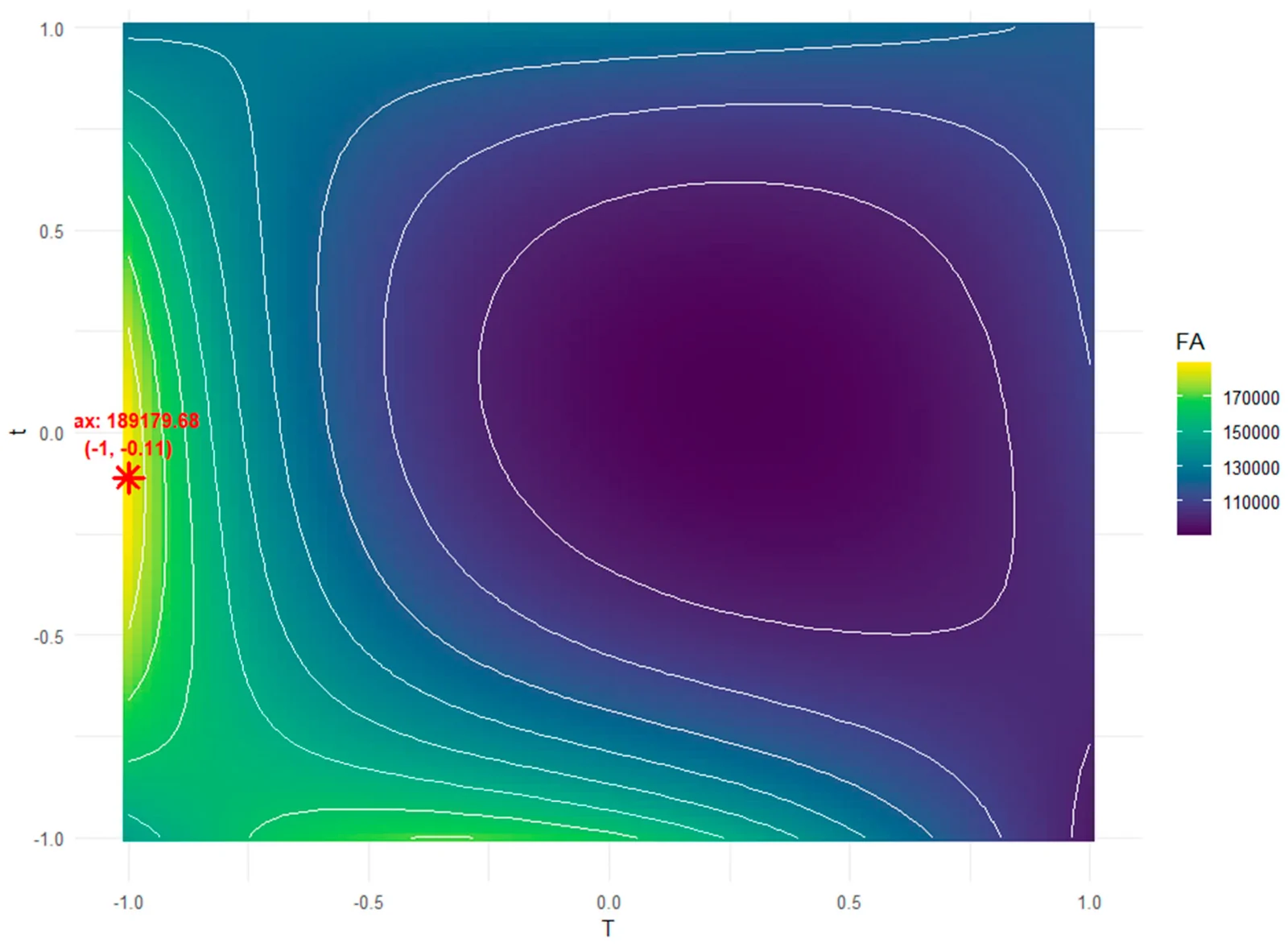
2D contour plot of the fitted response surface for 2,3,4,5,6-pentafluorobenzyl formate (FA). The factor values on the X and Y axes are expressed in coded variables for T, temperature, and t, time. The red marker denotes the maximum predicted response (y = 1.89·10^5^) located at the factor domain boundaries T = −1.0, and t = −0.11. The model order fitted was a 4th-order biquadratic polynomial; the response was transformed using the Box–Cox transformation with lambda = −0.8 (R^2^_adj_ = 0.9669, R^2^_pred_ = 0.9485).

The 27 experiments carried out during the study of the SCFA microwave-assisted derivatisation reaction allowed us to compute six multilinear regression models. The models were used to find the optimal compromise conditions for simultaneously derivatizing the six analytes investigated. Briefly, since formic acid was the most problematic analyte, its optimal derivatisation conditions, i.e., 2 min derivatisation time and subsequent SPME caption at 20 °C (see Figure 2), were adopted for all six SCFAs. The choice was convenient since only propionic acid and isovaleric acid showed optimal derivatisation conditions, but were slightly different, both in terms of subsequent caption temperature (~30 °C for both acids) and effective derivatisation time (1 min for propionic acid and 3 min for isovaleric acid). Table 1 reports the experimental areas for each SCFA derivative employed to compute the regression models. Figure 2 illustrates the 2D contour plot of the fitted response surface for 2,3,4,5,6-pentafluorobenzyl formate. The maximum predicted response within the investigated experimental domain occurs at the design boundary (T at coded value −1.0, equal to 20 °C, and time at coded value −0.11, equal to 1.89 min). While the unconstrained stationary point appears to lie outside the evaluated factor space, extending the operating range below 20 °C or reducing reaction times below 2 min was not practical for the simultaneous extraction of the remaining analytes. Thus, these boundary conditions were accepted as an optimal compromise for the overall method.

To keep the main text focused on the analytical application rather than chemometrics, some statistical outputs and model evaluation metrics for 2,3,4,5,6-pentafluorobenzyl formate (FA) models are provided in the Supplementary Materials (Tables S1 and S2). As the scope of this work is analytical method development rather than chemometrics, comprehensive ANOVA tables and response surface evaluation metrics for all tested analytes are omitted from the manuscript but are available from the corresponding author upon reasonable request. Regarding the model selection, neither linear nor quadratic models were able to fit the data. Therefore, increasing the complexity of the model was considered to obtain an adequate description of the response in the experimental domain. For this reason, a nine-parameter fourth-order biquadratic model was fitted for the data of all analytes. It is recognised that fitting a model with as many parameters as design points requires caution, as such a model effectively fits the responses, leaving no degrees of freedom. However, because our design included true experimental replicates, we retained sufficient degrees of freedom to estimate the pure experimental error variance. Figure 2 shows the response surface contour plot for FA and reports some model diagnostic tools indicating that the fitted biquadratic model accurately captures the primary curvature of the response surface without exhibiting numerical instability or severe overparameterisation artefacts across the experimental domain. The primary objective of fitting biquadratic polynomials was empirical and descriptive rather than mechanistic or predictive. We used the models to smooth the response, map the factor-space topology, and generate response surface plots to visualise the region likely containing the optimum for each target analyte. We did not perform model validation using independent confirmation experiments because the models’ predicted optima fell within the tested experimental domain and strongly aligned with our expectations and empirical observations. Given that the fitted surface provided a consistent description of the trends within the investigated factor ranges, additional experimental runs were deemed redundant for the scope of this study.

After the derivatisation reaction, the derivatized solutions were briefly vortexed, transferred into pre-vacuumed vials, and analysed by GC-MS as described above. The results using MAD at 100 °C for 2 min were comparable to those using the conventional method discussed above.

### 2.4. Theoretical Fundamentals and Physicochemical Parameters of HS-SPME

The physicochemical properties of the PFBBr derivatives can provide a qualitative interpretation of their different behaviour under vacuum-assisted HS-SPME conditions. Since this study does not report experimental kinetic or equilibrium data for the SPME determination of SCFAs after derivatisation with PFBBr, the parameters discussed below are considered theoretical estimates and are used only to support the interpretation of the experimental results. As reported in Table 2, the estimated physicochemical properties (obtained by US Environmental Protection Agency’s EPI Suite [45]) of the PFBBr derivatives vary with the carbon chain length. In particular, the derivatives generally show decreasing vapour pressure and increasing log Kow as the chain length increases. These differences can affect both the transfer of the analytes into the headspace and their interaction with the SPME coating. The estimated Henry’s constants also indicate differences in the expected headspace partitioning behaviour among the derivatives [45,46,47]. However, these values should not be considered experimental partition coefficients or extraction constants.

The experimental results showed that vacuum assistance did not improve the response of all derivatives to the same extent. PFB-formate and PFB-acetate showed higher responses under no-Vac conditions, whereas PFB-propionate, PFB-butyrate, PFB-isovalerate and PFB-hexanoate showed a predominant response under Vac conditions. For the latter derivatives, the Vac/no-Vac response ratios were 7.1, 8.2, 9.3 and 10.7, respectively. Interestingly, the response enhancement generally increased with increasing boiling point, in agreement with the trend previously reported by Solomou et al. [48]. This behaviour suggests that vacuum assistance can particularly benefit the extraction of the higher-boiling derivatives, although the present data do not allow a quantitative relationship between vacuum, extraction kinetics and equilibrium partitioning to be established.

The estimated log K_ow_ values further indicate increasing hydrophobicity from PFB-formate to PFB-hexanoate. Derivatives with log K_ow_ > 4, corresponding approximately to PFB-butyrate, PFB-isovalerate and PFB-hexanoate, may show greater affinity for hydrophobic surfaces and, consequently, a greater tendency towards adsorption phenomena. Conversely, PFB-formate presents the lowest estimated hydrophobicity and partition affinity. These considerations are consistent with the different responses observed experimentally and provide a qualitative basis for the use of the MSPME configuration for PFB-formate, where increasing the number of fibres can contribute to improving sensitivity.

Overall, the estimated physicochemical parameters support the interpretation of the observed differences among the PFBBr derivatives, but they should not be regarded as experimentally determined kinetic or equilibrium constants. Their role in this study is therefore limited to providing a qualitative physicochemical framework for the experimental behaviour observed under Vac and no-Vac conditions [45,46,47,48,49].

### 2.5. Green Assessment of Microextraction Technologies

GAC aims to make analytical procedures more environmentally friendly and safer for humans. Consequently, specific criteria and tools are required to assess environmental sustainability. The quantity and toxicity of reagents, waste production, energy requirements, miniaturisation, etc., are just some of the many criteria taken into account when assessing the environmental sustainability of a method. Combining derivatisation with METs aligns with GAC by reducing solvent use and integrating derivatisation with extraction. This approach facilitates the analysis of polar, non-volatile compounds by converting them into hydrophobic, volatile analytes for efficient gas chromatography separation. Principles 6 and 8 of GAC state that unnecessary derivatisation should be avoided. The reaction with PFBBr is the clearest case in which this prescription cannot be honoured since carboxylic acids are not amenable to GC without conversion of their active hydrogens. The question is therefore not whether to derivatize, but how to do so at the lowest environmental and occupational cost. Following this guideline, we used the minimum possible amount of solvent to dispense the PFBBr into the aqueous solution, i.e., 20 µL of derivatisation acetone solution. The central argument is that derivatisation acquires its green character from the extraction technique to which it is coupled. METs are solventless or nearly, allowing thermal desorption directly into the GC inlet and providing high enrichment factors, thus satisfying the GAC requirements of solvent minimisation and waste reduction before any consideration of the reagent [50,51].

**Figure 3:**
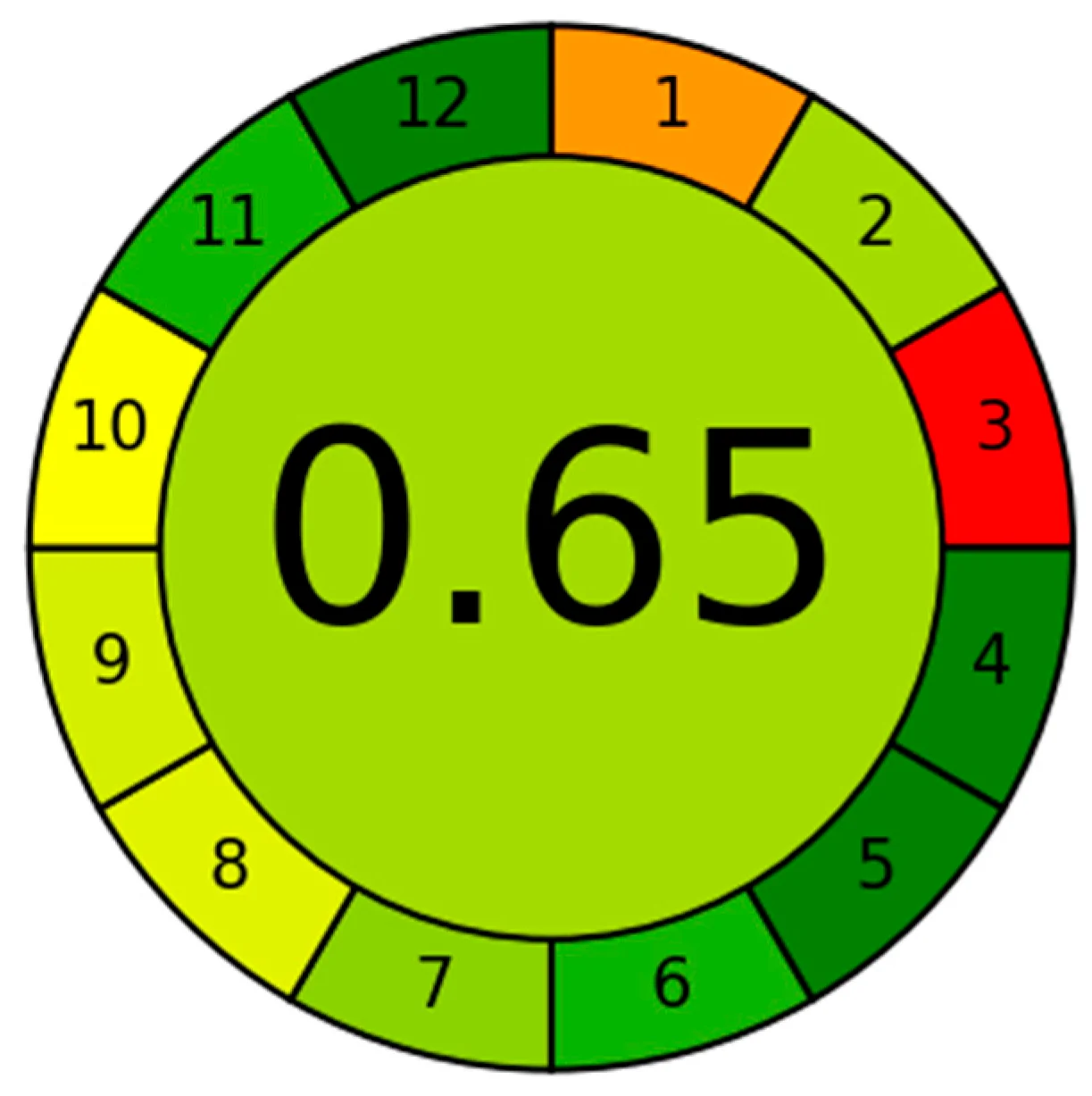
Greenness evaluation of the conventional GC method using AGREE software.

**Figure 4:**
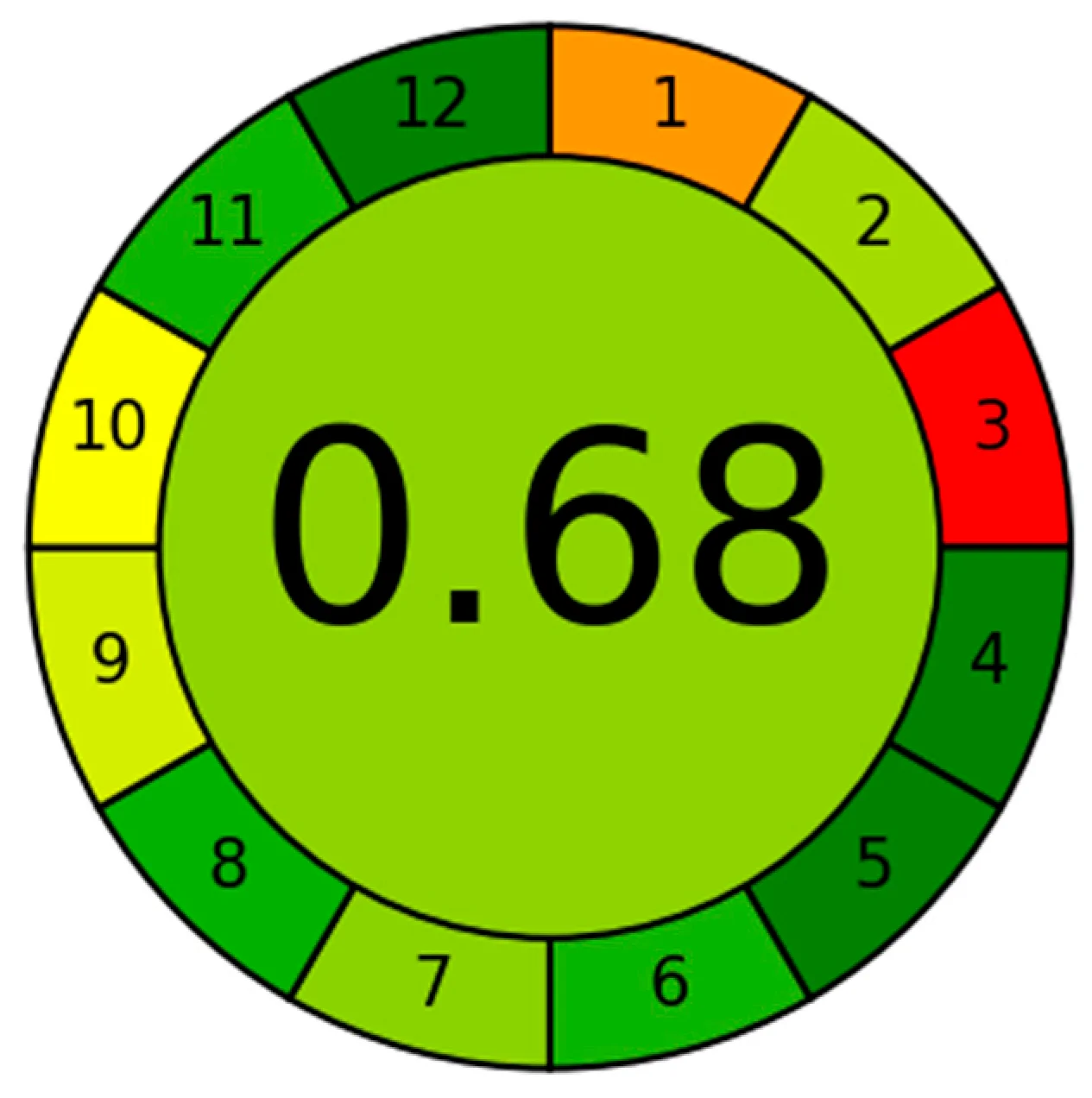
Greenness evaluation of the fast GC method using AGREE software.

In this work, we used an open-source calculator, called AGREE [52]: this software also takes into consideration energy consumption, expanding the binary nature of other software [50], thus overcoming the incorrect classification often observed [53]. The result for the conventional GC method is reported in Figure 3. The scores corresponding to GAC principles 1 and 3, representing sampling and subsequent pretreatment (despite the limited number of steps) and off-line analysis, respectively, are quite low. This aspect can unfortunately not be avoided, being considered the transport to the lab offline and the need for SCFA derivatisation. Moreover, principle 10 is slightly better, representing the bio-based origin of the reagents used which is only partially respected. Nonetheless, several aspects confirm the adherence to GAC principles, such as the number of steps, the degree of automation, and the operator’s safety (criteria 4, 5 and 12, respectively). The overall score of 0.65 confirms the greenness of the method proposed, despite the limitations which could not be avoided for the purposes of this work. As far as the fast GC-MS method is concerned, the AGREE software highlights a slight improvement in terms of compliance to GAC principles, as shown in Figure 4. In particular, principle 8, representing the number of analytes and the sample throughput, is now darker green: in fact, thanks to the reduced chromatographic run time and the fastest derivatisation procedure, the number of samples processed in one hour is substantially enhanced, from two to around six. The overall score for this method is 0.68, confirming the benefits introduced by the modifications proposed.

### 2.6. Method Performances and Paediatric Samples

In light of what is indicated above, the authors present the performance results for both conventional and fast GC methods in Table 3 and Table 4. The resulting calibration curves were linear in the investigated range for all the considered SCFAs, with correlation coefficients > 0.998 (the equations and R^2^ of each analyte are showed in the Supplementary Materials, Table S3). LOQs of the methods were in the 0.09–0.39 mg/L and 0.006–0.27 mg/L ranges, for conventional and fast GC methods, respectively. The precision of the assay estimated both as within- and inter-session repeatability resulted in the 2.8–7.1 and 1.2–6.3% ranges for the conventional GC method, and in the 3.6–5.7% and 2.5–6.1% ranges for the fast GC method, respectively. Accuracy was above 85% of the theoretical concentration, in line with the requirement of the US Food and Drug Administration for bioanalytical method validation (FDA’s Good Guidance Practices regulation). The slightly higher CVs% observed for the fast GC method proposed can be ascribed to the shorter sample preparation routine, in particular the shorter time employed for both equilibration and SPME caption; nonetheless, said results proved to be satisfactory for the aim of this work. As for carryover, Figures S1 and S4 of the Supplementary Materials show the chromatogram obtained after the analysis of the highest calibration level, i.e., 125 mg/L, for the conventional and fast GC method, respectively: as can be observed, there is a peak corresponding to the residual PFBBr. Following ICH M10 guidelines [54], the signal of each analyte in the blank sample analysed immediately after the calibration level should not exceed either 20% of the response for the analytes or 5% of the response of the IS. The carryover was calculated as 10% or less for both SCFA derivatives and the IS in the first blank sample. In the second and third blank samples subsequently analysed, said percentage was no longer quantifiable. For this reason, after each injection, a fibre bakeout step was implemented in the fully automated routine between chromatographic runs, to guarantee a complete absence of memory effect.

The recovery assessment was performed directly on faecal water obtained from the paediatric patients included in the study, thus evaluating the sample-preparation procedure in the authentic matrix subsequently analysed. Samples were fortified at 30 and 90 mg/L, and the endogenous concentration of each SCFA was determined in the corresponding unfortified sample and subtracted from the concentration measured in the fortified sample before calculating recovery [54]. This approach allowed the contribution of the naturally occurring SCFAs to be accounted for and the recovery of the added analyte to be assessed in the authentic biological matrix. The overall recovery values were higher than 86–90% for all investigated SCFAs with both the conventional and fast GC methods at the two concentration levels: no significant difference was observed comparing the determined amounts spiking the samples before or after the centrifugation and filtration steps. The fast GC method generally showed higher post-sample pretreatment recovery values, consistently reaching or exceeding 88–93% across the investigated analytes. Thus, comparison of samples fortified before and after centrifugation and filtration further indicated that these sample-processing steps did not result in relevant losses of the investigated SCFAs under the experimental conditions employed. The high and consistent recovery observed at both 30 and 90 mg/L supports the reliability of the sample-preparation procedure across the investigated concentration range. These results therefore provide direct evidence of satisfactory analyte recovery through the sample-preparation workflow in authentic patient-derived faecal water; in Table 3 and Table 4, the overall recovery is reported for each SCFA.

Although a conventional matrix-matched calibration could not be performed due to the lack of suitable matrix-free faecal water, the potential influence of the matrix was considered by evaluating the overall analytical performance of the method. The faecal samples underwent an initial 1:10 dilution, centrifugation and filtration, followed by further dilution during sample preparation, resulting in an overall 1:40 dilution of the original faecal material. This extensive dilution and clean-up step is expected to substantially reduce the concentration of endogenous matrix components potentially affecting derivatisation, HS-SPME partitioning or GC-MS detection. Moreover, the use of an internal standard further contributed to compensating for variability associated with sample preparation and instrumental response. An additional consideration is related to the nature of the SPME coating employed. The 85 µm polyacrylate (PA) fibre used in this study is an absorbent, liquid-like coating, in which analytes are extracted predominantly through partitioning into the polymeric phase rather than through adsorption onto a finite number of active surface sites. In contrast, porous adsorbent SPME coatings may be more susceptible to competition and displacement between analytes and matrix components because of their limited number of adsorption sites [55]. The use of a liquid-phase PA coating therefore further reduces the likelihood that competitive adsorption phenomena significantly affected the extraction of the investigated SCFAs under the experimental conditions employed. This feature, together with the extensive sample dilution and the use of an internal standard, provides additional support for the absence of a relevant matrix-related bias. Consistently, the method showed satisfactory accuracy for all investigated SCFAs, with values consistently above 85%, together with acceptable intra- and inter-day precision. Taken together, these results suggest that no relevant matrix-related bias was observed under the experimental conditions employed. Nevertheless, since a dedicated matrix-matched experiment was not performed, a residual matrix effect cannot be completely excluded and the present assessment should be considered indirect rather than a direct quantitative determination of matrix effect.

Figures S3–S5 report the chromatograms obtained using the fast GC-MS method developed for a calibration level, a fibre blank post-injection of said standard, and a paediatric sample.

The GC-MS method herein described was applied to human faecal water, which was used as a microbiota-derived luminal metabolite milieu to preliminarily investigate changes in SCFA concentrations before and after exposure to Caco-2 cells (Table 5). Table 6 reports the mean values and the standard deviation (SD) for each SCFA determined both before exposure with Caco-2 cells and after 24 h of incubation.

The present results confirm the presence of acetate, propionate and butyrate as the major SCFAs in human faecal-derived samples, in agreement with their predominance commonly reported in human faecal studies. For example, Huda-Faujan et al. [56] reported mean concentrations of 209.7, 93.3 and 176.0 µmol/g wet faeces for acetate, propionate and butyrate, respectively, in healthy subjects, while lower concentrations (162.0, 65.6 and 86.9 µmol/g wet faeces, respectively) were observed in patients with inflammatory bowel disease. These findings also illustrate the variability of faecal SCFA concentrations according to the study population and clinical condition. In the present study, SCFAs were determined in faecal water obtained after aqueous extraction and centrifugation, whereas most published clinical studies report concentrations in whole faeces, expressed per gram of wet or dry material. The two approaches therefore provide complementary information, and differences in faecal water content and sample preparation may contribute to the variability in the absolute concentrations measured. Although the present biological application is exploratory and was not designed to establish disease-specific alterations in SCFA profiles, it provides a proof of concept for the use of the proposed analytical platform to investigate SCFA patterns across physiological and pathological conditions and their potential interaction with intestinal epithelial cells. Nevertheless, the concentrations observed in the present samples are consistent with the presence of the major SCFAs at biologically relevant levels.

## 3. Materials and Methods

### 3.1. Faecal Sample Collection and Processing

Faecal samples were collected at home from paediatric patients who had a diagnosis of MASLD and from control, non-obese children without clinical or biochemical evidence of liver disease who were undergoing routine clinical care. The samples were sent to the Research Unit of Genetics of Complex Phenotypes at Bambino Gesù Children’s Hospital, IRCCS, Rome, following the principles of the Declaration of Helsinki. The study was approved by the Ethics Committee. Written informed consent was given by all the patients. The patients were told to keep samples at 4 °C. They were asked to bring the samples to the lab within 24 h of collecting them. Upon arrival, samples were either processed within 24 h or immediately frozen at −80 °C until processing. Briefly, 1 g of faecal sample was diluted 1:10 in 10 mL of ice-cold phosphate-buffered saline (PBS) (Merck, Darmstadt, Germany) and centrifuged at 13,000 rpm for 1 h; the centrifugation temperature was set at 4 °C. The resulting supernatant was collected, filtered through a 0.45 μm filter (Merck, Darmstadt, Germany), aliquoted, and stored at −80 °C until analysis. The processed samples obtained following this procedure were defined as faecal water. Then, for the in vitro experiments, faecal water was added to EMEM culture media supplemented with 1% Foetal Bovine Serum (FBS) (Merck, Darmstadt, Germany) with a 1:1 dilution and used to apically treat a monolayer of commercially available Caco-2 cells seeded on PET (polyethylene terephthalate) membrane inserts with 0.4 μm pores (Greiner Bio-One, Kremsmünster, Austria) after complete differentiation into enterocytes. Faecal-water-containing culture media were analysed at two experimental time points: immediately before exposure to Caco-2 cells (T0) and after 24 h of incubation with the cells (T24).

### 3.2. Chemical Reagents and Procedures

Fast Fit Assembly (FFA) 85 µm polyacrylate (PA) SPME fibres were purchased from Chromline (Prato, Italy). A Vac-SPME-Fiber kit (part no. 20-102) by ExtraTECH Analytical Solutions equipped with a conditioned/ready-to-use cylindrical ThermogreenTM LB-2 septa (part no. 20608 Supelco) was purchased from Markes International Ltd. (Bridgend, UK). 2-Ethylbutyric acid (CAS 88-09-5), formic acid (CAS 64-18-6), acetic acid (CAS 64-19-7), propionic acid (CAS 79-09-4), isovaleric acid (CAS 503-74-2), hexanoic acid (CAS 142-62-1) and PFBBr (CAS 1765-40-8) were purchased from Merck (Darmstadt, Germany). Materials were used for all experiments for closure of the 20 mL HS crimp-top vial (Markes International Ltd.). A SP-D Discover microwave by CEM Corporation (Matthews, NC, USA) was employed to perform PFBBr derivatisation at 100 °C for 2 min (operating at 300 W). A LA-BOPORT^®^ N820G (KNF Service GmbH, Freiburg im Breisgau, Germany) pumping unit (8 mbar vacuum without gas ballast) connected to an 18-gauge (1.219 mm external diameter) needle was used to evacuate the air inside the 20 mL HS vial.

### 3.3. Sample Preparation and Conventional GC Method

An aliquot of 250 µL faecal water sample was added to 750 µL of ultrapure water. The prepared sample was then supplemented with 50 µL of internal standard (2-ethylbutyric acid, 1 mg/L) and 20 µL of derivatisation solution (300 µL of PFBBr diluted to 1 mL with acetone), and subjected to derivatisation in a thermostated oven with agitation for 3 h at 60 °C. Extraction was performed via HS-SPME using a PA fibre for 20 min, following a 20 min equilibration period at 60 °C.

### 3.4. Microwave Derivatisation

For fast GC analysis the samples were prepared as described for the conventional GC method; the derivatisation was performed for 2 min at 100 °C using the microwave setup described above. Then, liquid extracts were introduced into pre-vacuumed 20 mL vials; after 2 min of equilibrium at 90 °C, SPME caption was performed for 2 min in cooling mode (20 °C), followed by 2 min desorption in the GC inlet. When using multiple extraction mode, the cycle was repeated on a vial containing the second aliquot of the same sample using the same SPME fibre, with subsequent single desorption.

### 3.5. Calibration Curves

A working solution containing SCFA mix at 1000 mg/L was prepared daily in ultrapure water. Following the derivation routines described above, two sets of seven-level calibration curves were prepared daily, for both conventional and fast GC methods. The concentrations of the standards were 1.0, 5.0, 10.0, 25.0, 50.0, 100.0 and 125.0 mg/L, respectively, adding proper amounts of the working solution to ultrapure water up to a volume of 1 mL.

### 3.6. Online Robotic System

**Figure 5:**
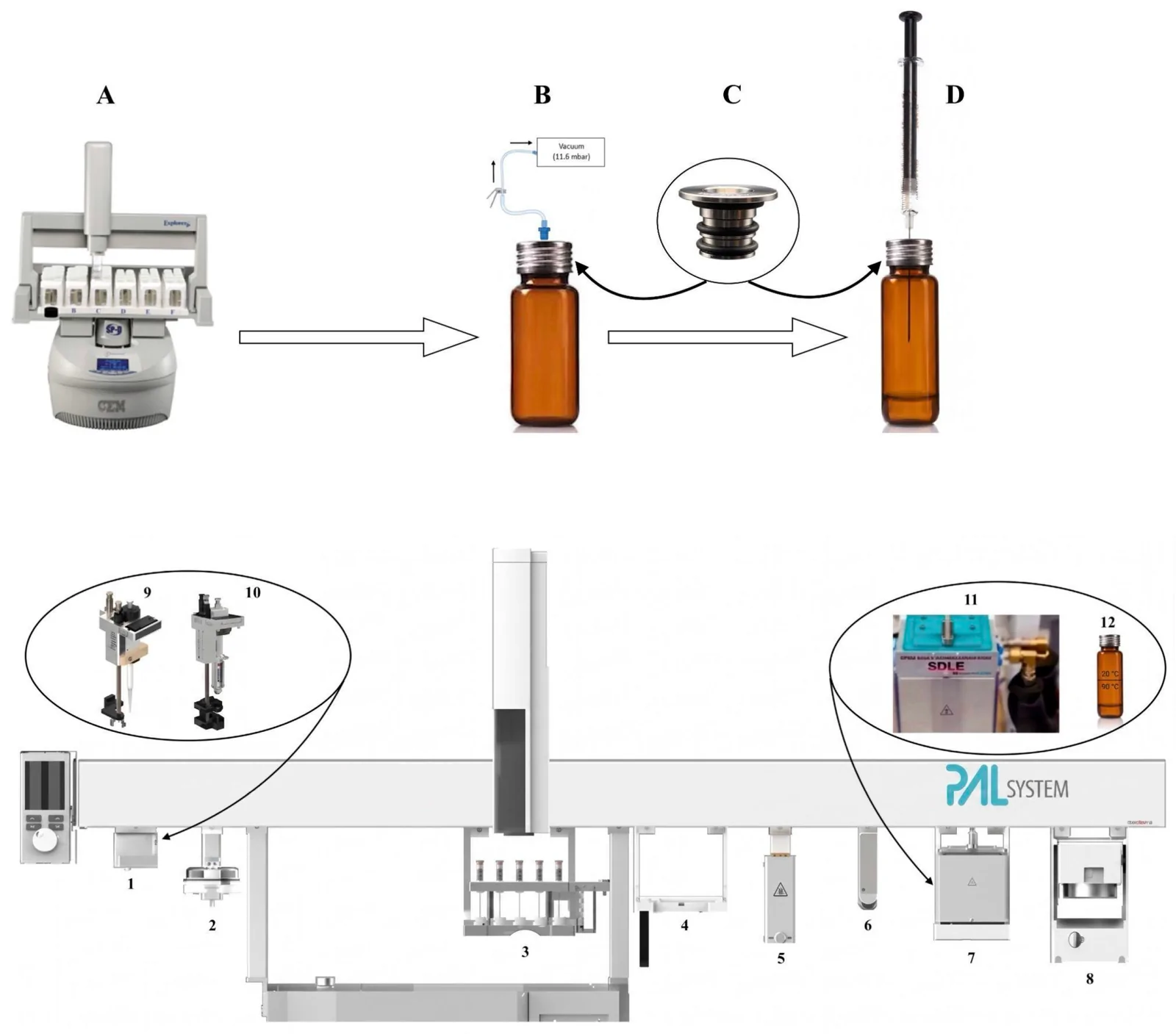
Autosampler employed for the fully automated procedure. 1. Park station; 2. wash/solvent modules; 3. FFA tray holder; 4. vial tray holder; 5. fibre conditioning station; 6. barcode reader; 7. agitator; 8. decapper; 9. Pipette tool; 10. MFX; 11–12. SDLE configuration; A. CEM microwave; B. LABOPORT N820G pumping unit; C; vacuum caps; D. extract dispensation in pre-vacuumed vials.

A CTC PAL3 RTC System xyz autosampler (CTC Analytics AG, Industrie Strasse 20 CH-4222, Zwingen, Switzerland) was employed for the full automation of the analytical routine. The system, composed of a 1600 mm bar, was equipped with the following tools (Figure 5): a park station, wash/solvent modules, a FFA tray holder, a 20 and a 2 mL tray (CTC Analytics AG, Zwingen, Switzerland), an SPME fibre conditioning system, a barcode reader, an agitator, a decapper module, two pipette tools (1000 and 100 μL, respectively), a Multi-Fibre eXchange (MFX) system and an SPME Dual-Layer Extractor (SDLE) developed by Chromline (Prato, Italy). The autosampler runs on Chronos software ver. 3.5 advance (Axel Semrau GmbH, Sprockhövel, Germany), enabling an efficient routine between reagent dispensation, the shift from syringe to 85 µm PA FFA-SPME fibre, the exchange of FFA-SPME fibres, the opening of the port for inserting the 10 mL Pyrex vial into the microwave synthesiser, the SPME sampling in cooling mode coupled with the vacuum-assisted technique, and the management of the single-fibre MSPME via the MFX [32,40]. Between every chromatographic run, the SPME fibre was conditioned in the bakeout station (250 °C, nitrogen flow ~20 mL/min) to guarantee an absence of memory effects. The fibre was used without any issue for around 120 samples.

### 3.7. GC-MS Analysis

A Shimadzu (Kyoto, Japan) GC2010/MS2010 equipped with two injector ports (290 °C and split ratio 20:1) and two GC analytical columns was used. For the conventional GC method, a Varian Factor FOUR VF-5 ms (60 m × 0.25 mm Internal Diameter (ID) × 1.0 µm film thickness) capillary column was employed (J&W Agilent Technologies, cat. no. CP8949, Santa Clara, CA, USA), operating at 40 °C for 5 min, then ramping to 220 °C (15 °C/min), then ramping to 320 °C at 20 °C/min, and held for 2 min (total programme time 24.00 min). For fast GC, a narrow-bore SLB5-MS customised column (5 m × 0.1 mm ID × 0.4 µm film thickness, Supelco, Merck, Milan, Italy) was held at 65 °C for 0.3 min, with a ramp of 80 °C/min up to 100 °C and 50 °C/min up to 275 °C (total programme time 5.24 min). EI-MS acquisition was performed in SCAN mode, with a 40–450 m/z mass range. Ion source and interface temperatures were set to 220 °C and 250 °C, respectively.

### 3.8. Design of Experiments and Data Analysis

The derivatisation experiments with PFBBr were studied applying the design-of-experiments approach (DoE). The responses considered were the SCFA peak areas. Two factors were evaluated, namely the SPME caption temperature (T, °C) and time (t, min). The ranges of these factors were 20–40 °C and 1–3 min, respectively. A canonical full factorial 3^2^ plan was adopted and every experiment was replicated three times independently. Data analysis and visualisation were performed using RStudio (Version 2026.08.1 Build 195; Posit Software version 2026.10.0, PBC, 2026).

### 3.9. Greenness Evaluation

The method proposed was evaluated by means of Analytical GREEnness Metric Approach and Software, whose latest version is available online [52].

It is a comprehensive, flexible, and straightforward assessment approach that provides an easily interpretable and informative result. The assessment criteria are taken from the 12 principles of green analytical chemistry and are transformed into a unified 0–1 scale. The final assessment result is the product of the assessment results for each principle. The output is a 12-section clock-like graph, with the overall score and colour representation in the middle. The performance of the procedure in each GAC principle is reflected with the intuitive red–yellow–green colour scale, while the weight of each principle is reflected with the width of its corresponding segment. The assessment can be easily performed using user-friendly software, with an automatically generated graph and an assessment report. The overall score is shown in the centre of the pictogram: values close to 1 and the colour dark green indicate that the procedure being assessed is more environmentally friendly. The performance of the procedure in each of the assessment criteria is reflected by the colour in the segment with the number corresponding to each criterion. For each principle, a different weight can be attributed, represented by the width of its section, while the central number represents the overall greenness score. The same weighting was attributed to the 12 principles, to obtain an easily comprehensible comparison between the methods surveyed.

### 3.10. Method Performance Evaluation

The performances of the method were estimated for both conventional and fast GC setups. Limits of detection (LODs) and quantification (LOQs) were calculated using the signal-to-noise (S/N) approach, applying S/N ratios of 3 and 10, respectively, in six different days, and the mean values were calculated. To evaluate the performances of the methods in terms of accuracy and precision, two in-house control solutions were prepared (at 30.0 and 90.0 mg/L, respectively) and processed following the derivatisation routine described above. In particular, inter-day performances were estimated by means of three distinct sets of calibration levels and control solutions, on six non-consecutive days; conversely, intra-day performances were monitored using six distinct sets of calibration levels and control solutions, processed sequentially. Each analyte was then quantified by means of peak-area ratio approach with an internal standardisation, plotted against its concentration, and a calibration curve was constructed for each SCFA PFBBr derivative. Least squares linear regression allowed us to find the best-fitting function. Coefficient of variation (CV%) was employed to express precision, by means of replicate analysis of control solutions, while accuracy was calculated using the yield of experimental concentrations divided by nominal amounts.

Carryover was evaluated by means of ICH M10 [54], injecting 3 blank samples (consisting of ultrapure water) after the highest calibration level at 125 mg/L, evaluating the response of both IS and analytes in six different days, and calculating the mean values.

The recovery of the sample-preparation procedure was evaluated using faecal water samples obtained from the paediatric patients included in the study. Since SCFAs are endogenous components of faecal water, the corresponding endogenous concentrations were determined in unfortified samples and taken into account in the recovery calculation. Aliquots of faecal water were fortified with the investigated SCFAs at two concentration levels, 30 and 90 mg/L. For each concentration level, the analytes were added before the centrifugation and filtration steps or after these sample-clean-up procedures, to evaluate both overall and post-sample pretreatment recovery. The samples were then subjected to the subsequent sample-preparation steps, including the required dilution and derivatisation procedures, and analysed by GC-MS under the conditions described above. Recovery was calculated by comparing the amount of analyte recovered after the complete analytical procedure with the nominal amount added to the sample, after subtraction of the corresponding endogenous concentration. Thus, recovery was calculated according to the following equation (Equation (1)):

(1) $$\mathrm{Recovery}\;\left(\%\right)=\;\frac{C_{\mathrm{spiked}}\;-\;C_{\mathrm{endogenous}}}{C_{\mathrm{added}}}\times 100$$

where *C_spiked_* is the concentration measured in the fortified sample, *C_endogenous_* is the concentration measured in the corresponding unfortified sample, and *C_added_* is the nominal concentration added. The comparison between samples fortified before and after centrifugation and filtration was used to assess the potential loss of analytes associated with these sample-processing steps.

The potential influence of the faecal water matrix on the analytical response was indirectly assessed from the method performance data. Faecal water samples were subjected to a 1:10 preliminary dilution, centrifugation and filtration, followed by the subsequent dilution steps required by the analytical procedure, resulting in an overall 1:40 dilution before analysis. Therefore, the residual matrix load in the final analytical solution was expected to be substantially reduced. In addition, the use of an internal standard and the evaluation of accuracy and precision were considered to provide complementary evidence of the absence of relevant matrix-related bias. Since matrix-free faecal water was not available, a conventional matrix-matched calibration approach could not be performed.

## 4. Conclusions

This study established a novel, high-throughput, and environmentally friendly analytical workflow for the profiling of SCFAs in human faecal water samples. By integrating microwave-assisted derivatisation with PFBBr, the traditionally time-consuming reaction phase was reduced from 3 h to 2 min. The optimisation of an automated Vac-HS-cooling-SPME system, combined with a single-fibre multiple-SPME configuration, significantly enhanced analyte mass transfer and extraction efficiency. This setup effectively resolved the low response typically associated with polar analytes, like formate. Coupling this streamline sample preparation to a fast GC-MS platform decreased total chromatographic run times from 24.00 to 5.24 min, tripling sample throughput from two to approximately six samples per hour. The method demonstrated excellent analytical performance across all target SCFAs, yielding strong linearity (R^2^ > 0.998), low limits of detection (0.002–0.09 mg/L), high overall recovery (86–90%), reliable precision (CV ≤ 6.1%), and accuracy (>85%). Furthermore, quantitative greenness evaluation using the AGREE metric confirmed that the fast GC-MS protocol improved the overall sustainability score from 0.65 to 0.68, aligning with green analytical chemistry principles by minimising solvent usage, energy consumption, and manual handling. Overall, this automated and eco-friendly methodology provides an efficient analytical tool for clinical and metabolomic laboratories studying SCFAs in both physiological and pathological subjects. Further experiments will be necessary to introduce the features of such impacts into the pathophysiology of specific diseases.

## Figures and Tables

**Table 1:** Experimental areas obtained for SCFA derivatives employed for method optimisation; HS temperature was studied at 20, 30 and 40 °C, while derivatisation time was studied at 1, 2 and 3 min, respectively.

		2,3,4,5,6-Pentafluorobenzyl formate			2,3,4,5,6-Pentafluorobenzyl acetate			2,3,4,5,6-Pentafluorobenzyl propionate		
20 °C	1 min	141,117	145,783	150,448	113,642	117,399	121,155	106,729	110,258	113,786
	2 min	178,417	188,602	198,786	157,959	166,976	175,992	98,146	103,749	109,351
	3 min	125383	127,942	130,500	130,194	132,851	135,508	144,445	147,393	150,340
30 °C	1 min	156,173	162,850	169,526	173,018	180,416	187,813	186,784	194,770	202,755
	2 min	87,031	93,181	99,330	98,220	105,161	112,101	88,583	94,843	101,102
	3 min	125,876	127,664	129,451	128,094	129,913	131,731	129,500	131,339	133,177
40 °C	1 min	93,742	97,547	101,351	94,167	97,989	101,810	94,152	97,973	101,793
	2 min	106,246	108,304	110,361	120,397	122,729	125,060	122,142	124,508	126,873
	3 min	114,882	118,070	121,257	113,896	117,057	120,217	114,805	117,991	121,176
		2,3,4,5,6-Pentafluorobenzyl butyrate			2,3,4,5,6-Pentafluorobenzyl isovalerate			2,3,4,5,6-Pentafluorobenzyl hexanoate		
20 °C	1 min	145,517	150,328	155,138	99,254	102,536	105,817	90,263	93,247	96,230
	2 min	139,411	147,369	155,326	144,205	152,437	160,668	103,297	109,194	115,090
	3 min	156,165	159,352	162,539	128,997	131,630	134,262	150,918	153,998	157,078
30 °C	1 min	140,115	146,106	152,096	138,845	144,782	150,718	125,167	130,519	135,870
	2 min	93,472	100,078	106,683	93,328	99,923	106,517	96,285	103,089	109,892
	3 min	137,328	139,278	141,227	143,522	145,560	147,597	147,243	149,334	151,424
40 °C	1 min	95,026	98,883	102,739	93,791	97,598	101,404	93,865	97,675	101,484
	2 min	111,756	113,921	116,085	119,924	122,247	124,569	111,974	114,143	116,311
	3 min	114,556	117,735	120,913	114,032	117,197	120,361	115,057	118,250	121,442

**Table 2:** Physicochemical properties of the SCFA derivatives surveyed. * EPA EPI Suite (HENRYWIN); ** MPBPWIN EPA EPI Suite EPA; *** MPBPWIN EPA EPI Suite EPA.

Name	Molecular Weight g/mol	Henry Costant * atm·m^3^/mol	Vapour Pressure ** Pa	Boiling Point *** 760 mmHg °C	Octanol–Water Partition Coefficients (log K_ow_) 25 °C
2,3,4,5,6-Pentafluorobenzyl formate	226	~1.05 × 10^−5^	~126.6	~189	2.73
2,3,4,5,6-Pentafluorobenzyl acetate	240	~5.13 × 10^−6^	~48	~211	3.22
2,3,4,5,6-Pentafluorobenzyl propionate	254	~2.51 × 10^−6^	~17.3	~232	3.71
2,3,4,5,6-Pentafluorobenzyl butyrate	268	~1.23 × 10^−6^	~6.4	~252	4.20
2,3,4,5,6-Pentafluorobenzyl isovalerate	282	~1.15 × 10^−4^	~6.0	~246	4.43
2,3,4,5,6-Pentafluorobenzyl hexanoate	296	~2.94 × 10^−7^	~0.8	~292	5.19

**Table 3:** LOD, LOQ, intra- and inter-day precision, intra- and inter-day accuracy, and overall recovery (R) at 30 and 90 mg/L of the conventional GC method for SCFA determination.

SCFAs	LOD (mg/L)	LOQ (mg/L)	Precision (%)		Accuracy (%)		R (%)	R (%)
			Intra-Day	Inter-Day	Intra-Day	Inter-Day	(30 mg/L)	(90 mg/L)
2,3,4,5,6-Pentafluorobenzyl formate	0.05	0.15	5.6	4.7	88.7	87.1	88	90
2,3,4,5,6-Pentafluorobenzyl acetate	0.13	0.39	7.1	6.3	86.6	85.7	86	88
2,3,4,5,6-Pentafluorobenzyl propionate	0.08	0.24	5.2	5.7	91.5	90.5	89	89
2,3,4,5,6-Pentafluorobenzyl butyrate	0.10	0.30	4.1	2.9	90.3	89.8	89	88
2,3,4,5,6-Pentafluorobenzyl isovalerate	0.08	0.24	6.9	3.3	95.2	93.0	88	87
2,3,4,5,6-Pentafluorobenzyl hexanoate	0.03	0.09	2.8	1.2	96.4	94.7	89	89

**Table 4:** LOD, LOQ, intra- and inter-day precision, intra- and inter-day accuracy, and overall recovery (R) at 30 and 90 mg/L of the fast GC method for SCFA determination.

SCFAs	LOD (mg/L)	LOQ (mg/L)	Precision (%)		Accuracy (%)		R (%)	R (%)
			Intra-Day	Inter-Day	Intra-Day	Inter-Day	(30 mg/L)	(90 mg/L)
2,3,4,5,6-Pentafluorobenzyl formate	0.006	0.018	4.9	4.2	87.1	85.1	88	90
2,3,4,5,6-Pentafluorobenzyl acetate	0.090	0.27	5.7	6.0	88.6	86.0	87	88
2,3,4,5,6-Pentafluorobenzyl propionate	0.060	0.18	5.4	5.2	90.7	94.2	89	89
2,3,4,5,6-Pentafluorobenzyl butyrate	0.014	0.042	4.9	3.3	90.4	91.2	90	90
2,3,4,5,6-Pentafluorobenzyl isovalerate	0.021	0.063	5.4	6.1	93.4	94.4	90	90
2,3,4,5,6-Pentafluorobenzyl hexanoate	0.002	0.006	3.6	2.5	92.8	96.6	90	88

**Table 5:** Determination of SCFAs in faecal water, extracted from stools of both paediatric patients affected by MASLD and control samples, analysed before exposure to Caco-2 cells (pink rows) and after 24 h of incubation with the cells (green rows), respectively.

ID	Group	Time (h)	2,3,4,5,6-Pentafluorobenzyl Formate (mg/L)	2,3,4,5,6-Pentafluorobenzyl Acetate (mg/L)	2,3,4,5,6-Pentafluorobenzyl Propionate (mg/L)	2,3,4,5,6-Pentafluorobenzyl Butyrate (mg/L)	2,3,4,5,6-Pentafluorobenzyl Isovalerate (mg/L)	2,3,4,5,6-Pentafluorobenzyl Hexanoate (mg/L)
ID_40	CTRL	0	<LOQ	4.323	1.663	0.666	0.279	0.025
ID_39	CTRL	0	<LOQ	4.546	1.600	0.670	0.234	<LOQ
ID_42	CTRL	0	0.040	4.799	3.254	1.991	0.250	<LOQ
ID_47	CTRL	0	<LOQ	1.532	8.334	5.902	0.568	<LOQ
ID_46	CTRL	0	<LOQ	8.287	2.562	4.587	0.209	<LOQ
ID_36	MASLD	0	<LOQ	23.193	24.280	7.693	0.107	0.012
ID_38	MASLD	0	<LOQ	9.142	2.270	4.103	<LOQ	<LOQ
ID_37	MASLD	0	0.029	24.017	11.996	5.389	0.368	0.012
ID_41	MASLD	0	<LOQ	10.436	9.644	7.316	0.808	0.011
ID_43	MASLD	0	0.075	17.232	11.022	6.610	0.182	<LOQ
ID_44	MASLD	0	<LOQ	9.946	4.912	2.162	0.264	<LOQ
ID_45	MASLD	0	0.058	18.778	9.366	13.972	0.888	0.011
ID_81	MASLD	0	<LOQ	<LOQ	<LOQ	1.45	<LOQ	1.02
ID_82	MASLD	0	0.03	3.46	1.45	1.93	0.45	<LOQ
ID_83	MASLD	0	0.04	2.45	1.11	5.71	0.44	1.55
ID_62	CTRL	24	<LOQ	2.069	0.327	<LOQ	<LOQ	0.015
ID_58	CTRL	24	<LOQ	8.497	1.551	0.050	<LOQ	0.010
ID_63	CTRL	24	<LOQ	1.328	0.188	<LOQ	<LOQ	0.009
ID_59	CTRL	24	<LOQ	3.939	0.676	0.056	<LOQ	<LOQ
ID_69	CTRL	24	<LOQ	7.645	3.443	0.708	0.215	0.014
ID_77	CTRL	24	0.049	14.145	3.550	2.703	0.368	0.024
ID_79	CTRL	24	<LOQ	5.385	1.254	1.028	0.108	0.009
ID_70	CTRL	24	0.022	4.816	2.108	0.558	0.169	<LOQ
ID_78	CTRL	24	<LOQ	5.385	1.254	1.028	0.108	0.009
ID_80	CTRL	24	0.027	13.915	5.143	2.530	0.409	0.018
ID_52	MASLD	24	<LOQ	1.314	8.602	0.573	<LOQ	<LOQ
ID_56	MASLD	24	<LOQ	5.035	0.633	<LOQ	<LOQ	<LOQ
ID_54	MASLD	24	0.047	1.180	3.841	0.362	0.085	0.021
ID_60	MASLD	24	<LOQ	27.484	4.435	0.077	<LOQ	0.009
ID_53	MASLD	24	0.037	1.368	9.161	0.638	0.069	<LOQ
ID_57	MASLD	24	<LOQ	3.327	0.620	<LOQ	<LOQ	<LOQ
ID_55	MASLD	24	<LOQ	5.458	2.412	0.364	0.109	<LOQ
ID_61	MASLD	24	0.034	10.088	1.335	<LOQ	<LOQ	<LOQ
ID_67	MASLD	24	<LOQ	9.895	5.276	2.762	0.553	0.027
ID_71	MASLD	24	0.052	23.923	9.726	4.831	0.253	0.026
ID_73	MASLD	24	<LOQ	4.100	1.519	0.095	0.069	<LOQ
ID_75	MASLD	24	0.037	12.416	4.200	7.441	0.636	0.013
ID_68	MASLD	24	<LOQ	5.320	3.034	1.829	0.339	0.011
ID_72	MASLD	24	0.021	16.504	7.044	3.596	0.187	0.013
ID_74	MASLD	24	0.026	7.717	2.904	0.152	0.183	0.019
ID_76	MASLD	24	<LOQ	10.770	3.623	6.253	0.541	0.012

Note. CTRL, control samples, collected from inpatients without liver disease; MASLD, samples collected from MASLD patients.

**Table 6:** Mean concentrations (mean ± SD) of SCFAs measured in faecal water before exposure to Caco-2 cells and after 24 h of incubation with Caco-2 cells. SCFAs were quantified as their corresponding 2,3,4,5,6-pentafluorobenzyl (PFB) derivatives.

SCFAs	Before Exposure to Caco-2 Cells (mg/L)	After 24 h Incubation with Caco-2 Cells (mg/L)
2,3,4,5,6-Pentafluorobenzyl formate	0.02 ± 0.03	0.01 ± 0.02
2,3,4,5,6-Pentafluorobenzyl acetate	9.48 ± 7.84	819 ± 6.72
2,3,4,5,6-Pentafluorobenzyl propionate	6.23 ± 6.42	3.38 ± 2.74
2,3,4,5,6-Pentafluorobenzyl butyrate	4.68 ± 3.52	1.45 ± 2.05
2,3,4,5,6-Pentafluorobenzyl isovalerate	0.34 ± 0.26	0.17 ± 0.19
2,3,4,5,6-Pentafluorobenzyl hexanoate	0.18 ± 0.46	0.01 ± 0.01

## Data Availability

The data presented in this study are available on request from the corresponding author due to ethical restrictions.

## Supplementary Materials

The following supporting information can be downloaded at https://www.mdpi.com/article/10.3390/molecules31203716/s1, Table S1—Lack-of-Fit of the original response, 2,3,4,5-Pentafluorobenzylformate peak area; Table S2—Diagnostics and model selection metrics for transformed response surface candidate models. Response: 2,3,4,5-Pentafluorobenzylformate peak area, Box-Cox transformation using lambda = −0.8; Table S3—Calibration curve equations and coefficients of correlation (R^2^) obtained for each analyte during method validation by conventional and fast GC–MS; Figure S1—Chromatogram in TIC mode obtained using VF-5 60m column for SCFAs determined (Fiber blank post 125 mg/L calibration level). Only residual PFBBr is visible (~17.4 min); Figure S2—Chromatogram in TIC mode obtained using VF-5 60m column for SCFAs determined (Sample). 1. Formic derivative; 2. Residual PFBBr; 3. Acetic derivative; 4. Propionic derivative; 5. Butyric derivative; 6. Isovaleric derivative; 7. IS derivative; 8. Hexanoic derivative; Figure S3—Chromatogram in TIC mode obtained using fast GC method for SCFAs determined (100 mg/L standard). 1. Formic derivative; 2. Residual PFBBr; 3. Acetic derivative; 4. Propionic derivative; 5. Butyric derivative; 6. Isovaleric derivative; 7. IS derivative; 8. Hexanoic derivative; Figure S4—Chromatogram in TIC mode obtained using fast GC method for SCFAs determined (Fiber blank post 125 mg/L calibration level). Only residual PFBBr is visible (~2.10 min); Figure S5—Chromatogram in TIC mode obtained using fast GC method for SCFAs determined (Sample). 1. Formic derivative; 2. Residual PFBBr; 3. Acetic derivative; 4. Propionic derivative; 5. Butyric derivative; 6. IS derivative.

## Abbreviations

The following abbreviations are used in this manuscript:

AGREE	Analytical GREEnness Metric Approach and Software
CAS	Chemical Abstracts Service
CBS	Coated Blade Spray
EI-MS	Electron Ionisation Mass Spectrometry
EMEM	Eagle’s Minimum Essential Medium
ESI	Electrospray Ionisation
FFA	Fast Fit Assembly
GAC	Green Analytical Chemistry
HS-SPME	Headspace Solid-Phase Microextraction
ITEX	In-Tube Extraction
LLE	Liquid–Liquid Extraction
MAD	Microwave-Assisted Derivatisation
MASLD	Metabolic Dysfunction-Associated Steatotic Liver Disease
MEPS	Microextraction by Packed Sorbent
MFX	Multi-Fibre eXchange
µSPEed	Micro Solid-Phase Extraction Cartridges
NEMI	National Environmental Methods Index
PBTo	Persistent, Bioaccumulative, and Toxic
PFBBr	2,3,4,5,6-Pentafluorobenzyl bromide
TIC	Total Ion Chromatogram
Vac-HS-cooling	Vacuum-Assisted Headspace Cooling
MSPME	Multiple Solid-Phase Microextraction
VOCs	Volatile Organic Compounds
