# Supplementary material for: Rethinking Solid-Phase Microextraction (SPME) for Bioanalysis: Workflow Optimisation in Automated Vacuum-Assisted Cooling SPME for Fatty Acid Monitoring in Faecal Waters
Source: Molecules. 2026 Oct 16;31(20):3716. doi: 10.3390/molecules31203716 (PMC13707552; doi:10.3390/molecules31203716)
Supplement: Supplementary file 1 [file molecules-31-03716-s001.zip › molecules-4574391-supplementary.pdf]

Supplementary materials

Table S1 - Lack-of-Fit of the original response, 2,3,4,5-Pentafluorobenzylformate peak area.

Model	Parameters	p (LOF)	R^2adj	R^2pred	ΔR^2	AIC	BIC	p-value vs. Full
1. Simple Linear (no int.)	3	0	0.3511	0.2889	0.062	-518.71	-513.52	0
2. Linear with Interaction (T*t)	4	0	0.3990	0.3647	0.034	-519.93	-513.45	0
3. Quadratic (2nd Order)	6	0	0.3689	0.2017	0.167	-517.06	-507.99	0
4. Partial Cubic (3rd Order)	8	0	0.4453	0.2660	0.179	-519.25	-507.59	0
5. Full Biquadratic (4th Order)	9	1	0.9669	0.9485	0.018	-594.86	-581.90	NA

Table S2 - Diagnostics and model selection metrics for transformed response surface candidate models. Response: 2,3,4,5-Pentafluorobenzylformate peak area, Box-Cox transformation using lambda = -0.8.

Model	p	S	R^2	R^2 adj	R^2 pred	ΔR^2	p (LOF)	AIC	BIC
1. Simple Linear (no int.)	3	0.0000	0.4010	0.3511	0.2889	0.0621	0.0000	-518.71	-513.52
2. Linear with Interaction (T*t)	4	0.0000	0.4683	0.3990	0.3647	0.0343	0.0000	-519.93	-513.45
3. Quadratic (2nd Order)	6	0.0000	0.4902	0.3689	0.2017	0.1671	0.0000	-517.06	-507.99
4. Partial Cubic (3rd Order)	8	0.0000	0.5946	0.4453	0.2660	0.1793	0.0000	-519.25	-507.59
5. Full Biquadratic (4th Order)	9	0.0000	0.9771	0.9669	0.9485	0.0184	1.0000	-594.86	-581.90

Note: p = number of model parameters; S = residual standard error; R^2 adj = adjusted coefficient of determination; R^2 pred = predicted coefficient of determination based on PRESS; $\Delta R^2 = R^2adj - R^2pred$ (values > 0.20 indicate predictive overfitting); p (LOF) = Lack-of-Fit p-value against pure experimental error; AIC = Akaike Information Criterion; BIC = Bayesian Information Criterion.

Table S3 Calibration curve equations and coefficients of correlation (R^2) obtained for each analyte during method validation by conventional and fast GC–MS.

	Conventional GC-MS		Fast GC-MS	
Name	Equation	R^2	Equation	R^2
2,3,4,5,6-Pentafluorobenzyl formate	y = 0.0009x + 0.5352	0.998	y = 0.0008x + 0.5386	0.998
2,3,4,5,6-Pentafluorobenzyl acetate	y = 0.0008x + 0.7502	0.999	y = 0.0007x + 0.7594	0.998
2,3,4,5,6-Pentafluorobenzyl propionate	y = 0.0012x + 0.7805	0.998	y = 0.0011x + 0.804	0.999
2,3,4,5,6-Pentafluorobenzyl butyrate	y = 0.0015x + 0.9022	0.998	y = 0.0011x + 0.9142	0.998

2,3,4,5,6-Pentafluorobenzyl isovalerate	y = 0.0018x + 0.7455	0.999	y = 0.0016x + 0.7547	0.998
2,3,4,5,6-Pentafluorobenzyl hexanoate	y = 0.0009x + 0.7504	0.998	y = 0.001x + 0.7458	0.998

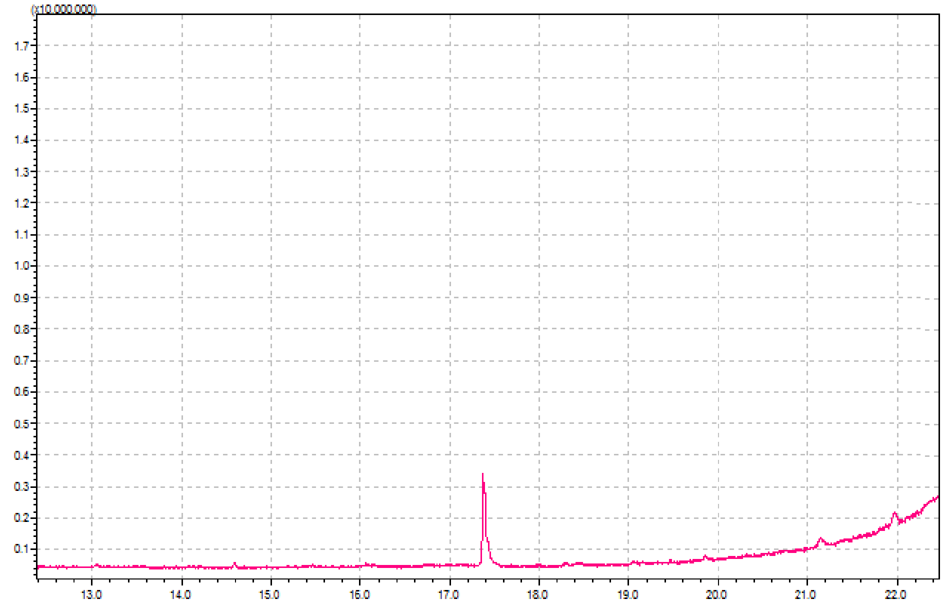

Figure S1 - Chromatogram in TIC mode obtained using VF-5 60m column for SCFAs determined (Fiber blank post 125 mg/L calibration level). Only residual PFBBr is visible (~17.4 min).

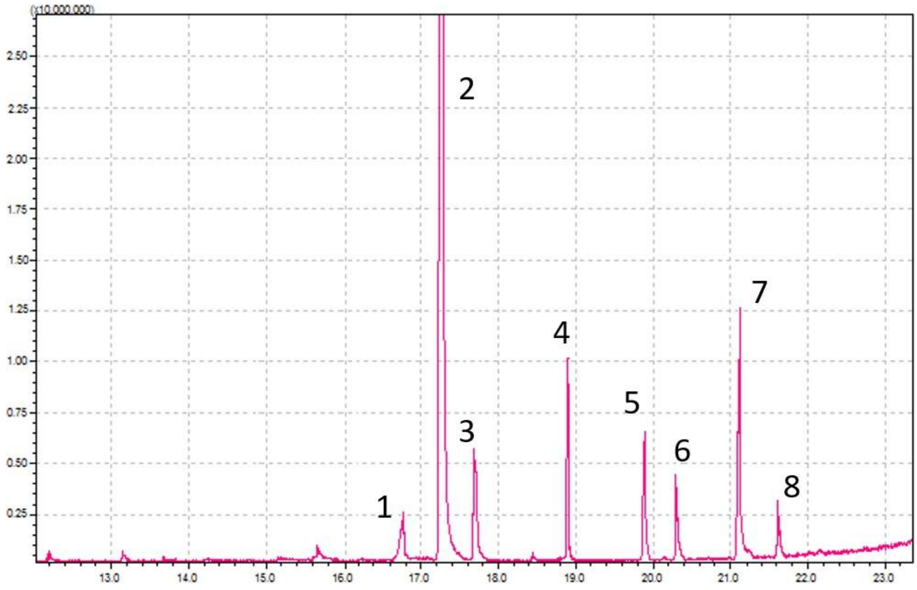

Figure S2 - Chromatogram in TIC mode obtained using VF-5 60m column for SCFAs determined (Sample). 1. Formic derivative; 2. Residual PFBBr; 3. Acetic derivative; 4. Propionic derivative; 5. Butyric derivative; 6. Isovaleric derivative; 7. IS derivative; 8. Hexanoic derivative.

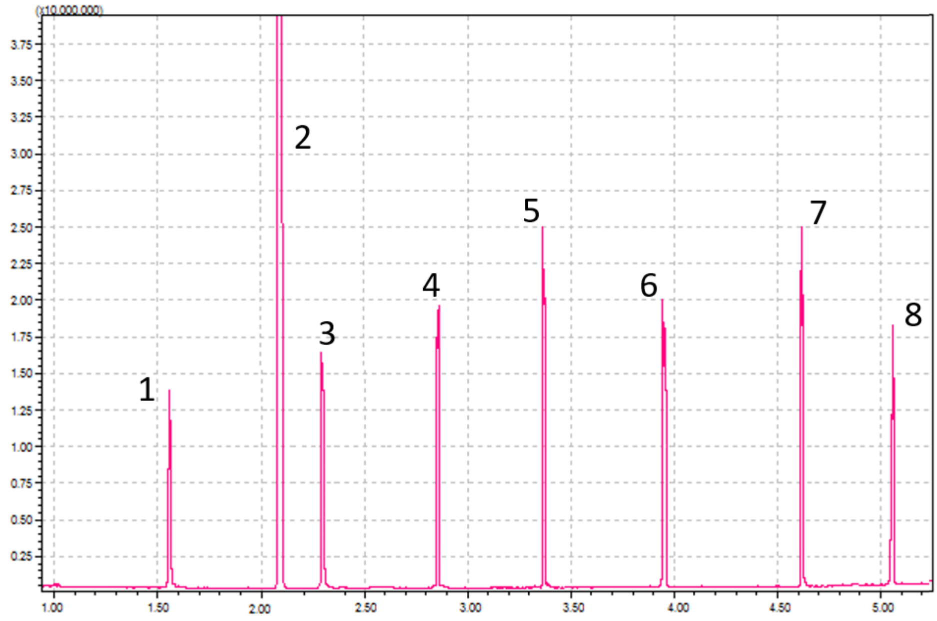

Figure S3 - Chromatogram in TIC mode obtained using fast GC method for SCFAs determined (100 mg/L standard). 1. Formic derivative; 2. Residual PFBBr; 3. Acetic derivative; 4. Propionic derivative; 5. Butyric derivative; 6. Isovaleric derivative; 7. IS derivative; 8. Hexanoic derivative.

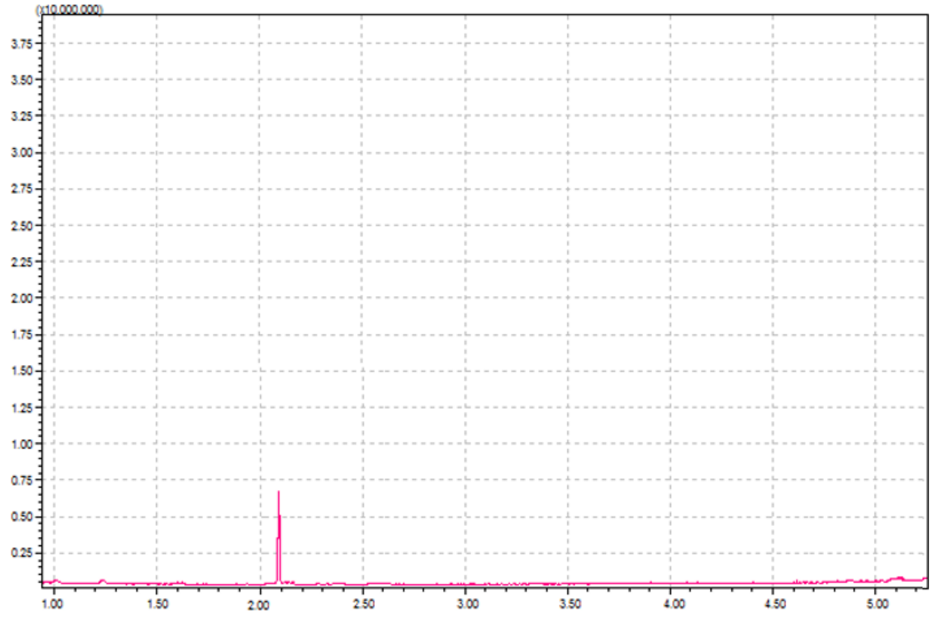

Figure S4 - Chromatogram in TIC mode obtained using fast GC method for SCFAs determined (Fiber blank post 125 mg/L calibration level). Only residual PFBBr is visible (~2.10 min).

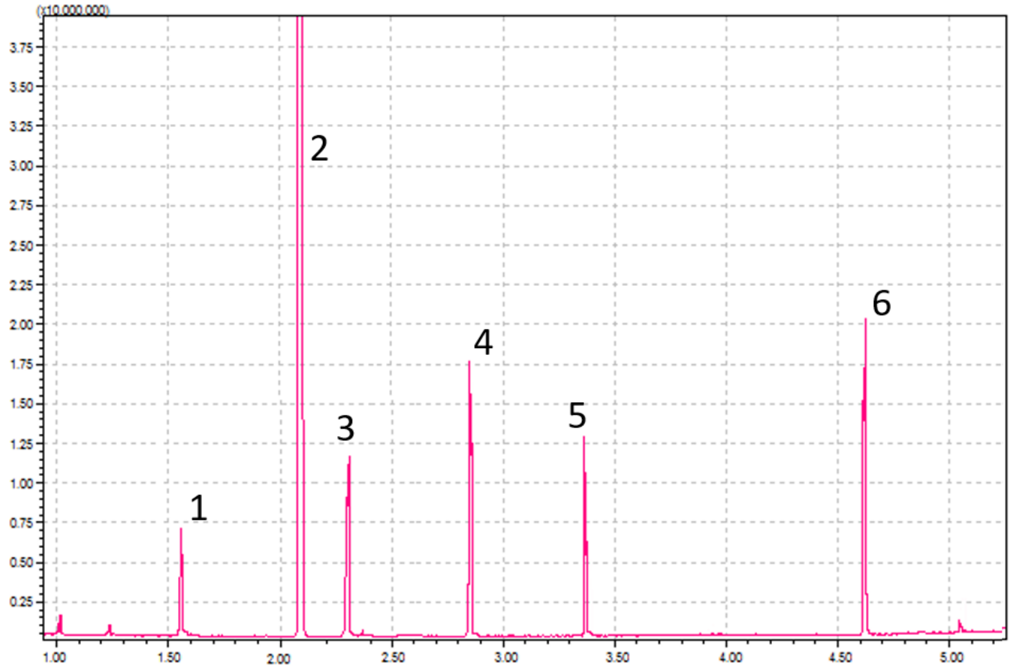

Figure S5 - Chromatogram in TIC mode obtained using fast GC method for SCFAs determined (Sample). 1. Formic derivative; 2. Residual PFBBr; 3. Acetic derivative; 4. Propionic derivative; 5. Butyric derivative; 6. IS derivative.
